# Effects of ReadiSorb L-GSH in Altering Granulomatous Responses against *Mycobacterium tuberculosis* Infection

**DOI:** 10.3390/jcm7030040

**Published:** 2018-03-01

**Authors:** Hicret Islamoglu, Ruoqiong Cao, Garrett Teskey, Karo Gyurjian, Sebastian Lucar, Marcel P. Fraix, Airani Sathananthan, John K. Chan, Vishwanath Venketaraman

**Affiliations:** 1 Department of Biological Sciences, California State Polytechnic University, Pomona, CA 91768, USA; hislamoglu@westernu.edu (H.I.); jkchan@cpp.edu (J.K.C.); 2 College of life Sciences, Hebei University, Baoding 071000, China; rcao@westernu.edu; 3 Department of Basic Medical Sciences, College of Osteopathic Medicine of the Pacific, Western University of Health Sciences, Pomona, CA 91766-1854, USA; kgyurdzhyan@westernu.edu (K.G.); asathananthan@westernu.edu (A.S.); 4 Graduate College of Biomedical Sciences, Western University of Health Sciences, Pomona, CA 91766-1854, USA; gteskey@westernu.edu; 5 College of Dental Medicine, Western University of Health Sciences, Pomona, CA 91766-1854, USA; sebastian.lucar@westernu.edu; 6 Departments of Physical Medicine and Rehabilitation and Neuromusculoskeletal Medicine/Osteopathic Manipulative Medicine, Western University of Health Sciences College of Osteopathic Medicine of the Pacific, Pomona, CA 91766-1854, USA; mfraix@westernu.edu

**Keywords:** *M. tb*, Mycobacterium tuberculosis, cytokines, glutathione, granuloma

## Abstract

*Mycobacterium tuberculosis* (*M. tb*), a rod-shaped acid-fast bacterium, is the causative agent of tuberculosis (TB). TB remains one of the leading causes of morbidity and mortality worldwide. Additionally, approximately one-third of the world’s population has latent tuberculosis infection (LTBI) as a result of the body’s primary mechanism of defense against *M. tb* infection, the formation of a granuloma. A granuloma is the aggregation of immune cells that encapsulate the bacteria to keep them localized to prevent further infection and thus the bacteria become quiescent. However, if an individual becomes immunocompromised, they become more susceptible to *M. tb*, which may lead to bacterial reactivation and an active infection, because the host is no longer able to generate adequate immune responses. In this study, we examined liposomal glutathione’s (L-GSH) effectiveness in promoting the formation of solid, stable granulomas. We assessed this ability by generating in vitro human granulomas constructed from peripheral blood mononuclear cells (PBMCs) that were derived from healthy subjects and testing their granulomatous effector responses against both *M. bovis* bacille Calmette–Guérin (BCG) and the highly virulent Erdman strain of *M. tb*. Additionally, we measured the survival and immune characteristics of the Erdman strain of *M. tb* in THP-1 originated macrophages as well as in vitro granulomas generated from individuals from type 2 diabetes (T2DM). Our results demonstrate that L-GSH treatment can decrease the intracellular survival of both BCG and virulent *M. tb*, as well as downregulate the levels of overexpressed proinflammatory cytokines delegated from the granulomas derived from not only healthy subjects but also individuals with T2DM.

## 1. Introduction

The World Health Organization reported that in 2015, approximately 10.4 million people were infected with *M. tb*, and of those 1.8 million cases were fatal. Poor health care access or individuals becoming immunocompromised, such as those who are elderly, have a history of substance abuse, contract HIV, or develop diabetes mellitus, are the prominent contributors to the increasing cases of TB through recent years [1]. Additionally, in 2015, the Center for Disease Control and Prevention (CDC) estimated that 30.3 million people in the United States had Type 2 Diabetes [2]. T2DM is a disease typically characterized by elevated serum glucose, due to either diminished insulin production or insulin resistance [3]. Uncontrolled T2DM impairs cell-mediated immunity, which results in an increased susceptibility to *M. tb* infection. These individuals with T2DM often display a higher risk of relapse after treatment as well [4]. This risk is associated with chronic inflammation, which is marked by an increase in pro-inflammatory cytokines and a decrease in immunomodulatory cytokines [5].

Archetypally the majority of individuals infected with *M. tb* are able to control the infection and contain it within a granuloma, an aggregation of immune cells that encapsulates the bacteria keeping it localized. Since the bacteria have not been completely eradicated, the infection is subsequently described as latent and the individual is at risk of future reactivation if they become immunocompromised [6]. *M. tb*’s scheme of survival is to reside within the macrophages and dendritic cells that phagocytize them by blocking the effector mechanisms including phagosome maturation, thereby preventing the pathogen containing phagosome from fusing with lysosomes [7]. However, initiation of adaptive immune responses will result in the production of IFN-γ (a macrophage-activating cytokine) by CD4+ T cells and generation of perforin and granulysin by CD8+ T cells, leading to effective control over the *M. tb* infection [8,9]. In contrast, increased levels of the cytokines TGF-β and IL-10 can dampen the effector responses against *M. tb* infection [8,10]. Alternatively, reactive oxygen species (ROS) can significantly contribute to control over a *M. tb* infection, specifically during the initial stages of infection [11]. However, if oxidative stress extends beyond the initial stages of infection cellular damage naturally ensues, which when combined with a cytokine imbalance can predispose individuals to reactivation of *M. tb* and an active disease [12,13].

Glutathione (GSH), a biological antioxidant found in most human cells, plays a critical role in maintaining redox homeostasis. GSH exists in two forms: the reduced and the oxidized form. Reduced GSH (rGSH) possesses the antioxidant activity, whereas the oxidized form (GSSG) is the byproduct of the reaction neutralizing ROS. In a healthy individual roughly 90% of the GSH is in the reduced form and available to maintain cellular functions [14]. Our lab has previously demonstrated that rGSH has direct antimycobacterial effects and can enhance the functions of natural killer and T cells to control a *M. tb* infection [15]. GSH enhancement caused a significant increase in the expression of cytotoxic ligands such as FasL and CD40L on the cell surface of NK cells, leading to apoptosis of *M. tb*-infected monocytes and improved control of intracellular *M. tb* infection [15,16,17]. Increasing the levels of GSH in T cells augmented the production of IL-2 and IFN-γ, leading to superior control of *M. tb* infection inside the macrophages [18].

Furthermore, individuals with T2DM have been shown to have compromised levels of rGSH due to a deficiency in the synthesis of the de novo enzymes that are responsible for GSH synthesis and thus exhibit a systemic increase in the production of ROS [19]. This prompted our team to hypothesize that the compromised levels of rGSH in individuals with T2DM will impair the granulomatous responses against a *M. tb* infection. Therefore, we tested our hypothesis by performing in vitro infection studies in THP-1 cells and in granulomas generated from PBMCs derived from healthy subjects as well as individuals with T2DM. Our results indicate that L-GSH treatment promotes the formation of solid/stable granulomas, decreases the intracellular survival of *M. bovis* (BCG- an attenuated vaccine strain of mycobacteria) and the Erdman strain of *M. tb* (highly virulent strain of *M. tb*), as well as downregulates the levels of overexpressed proinflammatory cytokines from healthy subjects and those with T2DM.

## 2. Materials and Methods

### 2.1. THP-1 Cell Culture

THP-1 cell line (Human monocytic leukemia cell line) was cultured in RPMI (Sigma, St. Louis, MO, USA) and 10% fetal bovine serum (Sigma, St. Louis, MO, USA) and incubated in 37 °C at 5% CO_2_. For the assays, cells were collected from the flasks, centrifuged at 2000 rpm for 15 min, resuspended in RPMI containing 10% FBS and counted for cell numbers. THP-1 cells (2 × 10^5^/well) were distributed in poly-lysine (Sigma, St. Louis, MO, USA) coated 24-well tissue culture plates. Differentiation of THP-1 cells to macrophages was achieved by adding PMA (Phorbol 12-myristate 13-acetate-Sigma) at a concentration of 10 ng/mL.

### 2.2. BCG Cell Culture

BCG was grown in Middlebrook 7H9 broth media supplemented with albumin dextrose complex (ADC), an enrichment supplement used in cultivation of mycobacteria, at 37 °C. Optical density (absorbance) of the culture was checked periodically. Once the optical density of mycobacterial cultures reached between 0.5–0.8 nm at A600, bacterial cell suspension was centrifuged at 3000 rpm to sediment the bacteria. Bacterial pellet was resuspended in PBS. Bacterial clumps were disaggregated by vortexing five times with 3-mm glass beads (the duration of each vortexing cycle was 2 min, with a 1-min interval between each vortex cycle). Bacterial suspension was then filtered through a 5-μm Millipore syringe filter to remove any further clumps. Processed mycobacteria were serially diluted and plated on Middlebrook 7H11 agar media supplemented with ADC to determine the bacterial counts.

### 2.3. Culture of Erdmann Strain of M. tb

Erdmann strain of *M. tb* expressing green fluorescent protein (GFP) was obtained as a gift from Dr. Selvakumar Subbian at Rutgers New Jersey Medical School, Biomedical and Health Sciences. Erdmann strain of *M. tb* is similar to H37Rv (standard laboratory strain of *M. tb*) other than doubling faster and being more virulent [1]. Erdmann strain of *M. tb* (will henceforth be referred to as *M. tb*) was handled inside a biosafety level-3 facility (BSL-3) and cultured in 7H9 media supplied with ADC at 37 °C. *M. tb* was processed to disaggregate the clumps by methods similar to those described in the previous section.

### 2.4. Liposomal Glutathione

L-GSH (ReadiSorb Liposomal Glutathione) was provided by Dr. Frederick T. Guilford (Your Energy Systems, Palo Alto, CA, USA). L-GSH contains rGSH. The liposomes in this reagent used are derived from lecithin. The advantage of using L-GSH over N-acetyl cysteine (NAC-a GSH precursor) is the ready availability of rGSH to the immune cells. Since our previous studies have shown that individuals with T2DM have deficiency in the levels of enzymes that are responsible for the de novo synthesis of GSH [20], NAC treatment may not effectively restore the levels of GSH, and therefore the best approach to enhance the levels of rGSH will be to treat cells with L-GSH, which can be easily taken up by the cells, leading to rapid restoration in the levels of rGSH. We considered a 40 μM concentration of L-GSH as optimal for treatment based on previous findings from our standardization experiments.

### 2.5. THP-1 Macrophage Infection and Treatment

Differentiated macrophages were infected with processed *M. tb* at a multiplicity of infection or MOI (multiplicity of infection) of 0.1:1 (bacteria to macrophage ratio). Infected macrophages were incubated for 1 h and then washed three times with warm 1 × PBS to remove the unphagocytosed bacteria. Infected macrophages were then either sham-treated or received L-GSH (40 μM) administered at three equal intervals (1 h, 4 d, and 8 d post-infection) throughout the trial. The infected cells were maintained at 37 °C, 5% CO_2_ until they were terminated at 1 h and 12 d post-infection to determine the intracellular survival of *M. tb*.

### 2.6. Termination of Macrophages for CFUs Assay

Termination of infected macrophages was performed by collecting and storing the cell-free supernatants and lysing THP-1 cells using 250 μL of ice cold, sterile 1 × PBS. Cell lysates collected from the wells were vigorously vortexed and then subjected to freeze/thaw cycles to ensure complete lysis of macrophages. The collected lysates and supernatants were then diluted in sterile 1 × PBS and plated on a Middlebrook 7H11 medium enriched with ADC to evaluate *M. tb* survival inside the macrophages by counting the bacterial colonies.

### 2.7. Subject Recruitment

Participants for this study were recruited after obtaining signed informed consent. The protocols for the study involving healthy subjects and participants with T2DM were approved by the Institutional Review Board of Western University of Health Sciences. The sample size included five T2DM participants between the ages of 51 and 74 years, without any preference for race, ethnicity, or gender. Inclusion criteria for T2DM required that participants have a documented diagnosis through Dr. Airani Sathananthan at Western University College of Osteopathic Medicine of the Pacific and have A1C levels of higher than or equal to 7% (the range for our patients was 8–12%). Exclusion criteria included currently taking or had taken L-GSH within the last six months, was allergic to L-GSH and/or soy, had chemotherapy within the last year, was currently pregnant, lactating, or had been pregnant within the last six months; pregnancy was considered a reason for study termination.

A total of eight healthy participants (non-HIV) were also recruited between the ages of 21–28 as additional controls for measurement of baseline levels of cytokines, free radicals, and GSH, as well as microscopic studies. Healthy volunteers did not consume a placebo or L-GSH. Criteria for selection included no known disease that compromises the immune system and no prescription drugs taken at the time of the blood draw. After signing an informed consent form, 40 mL of blood was drawn from each participant.

### 2.8. Isolation of Plasma and Peripheral blood Mononuclear Cells

Plasma and peripheral blood mononuclear cells (PBMCs) were isolated from peripheral blood of T2DM subjects and healthy individuals by Ficoll-Paque (Sigma, St. Louis, MO, USA) density centrifugation. This procedure involves centrifugation of blood layered on Ficoll-Paque medium at a 1:1 ratio at 1800 rpm for 30 min. Plasma (the top layer) was collected and stored at −80 °C, while PBMCs (the third layer from the top) were further washed three times with 1X phosphate-buffered saline (PBS) and then resuspended in RPMI containing l-glutamine and 5% human AB serum.

### 2.9. Induction of Granulomas

PBMCs (6 × 10^5^/well) were plated on a 24-well tissue culture plate, pre-coated with 0.001% poly-l-lysine, and infected with *M. tb* or BCG at an MOI of 0.1:1. Infected PBMCs were then either sham-treated or received L-GSH (40 μM) administered at four equal intervals (1 h, 4 d, 8 d, and 12 d post-infection) throughout the trial. Infected granulomas were maintained at 37 °C with 5% CO_2_ for 15 days. Granulomas were terminated at eight days and 15 days post-infection to determine the effects of L-GSH in altering the granulomatous responses against *M. tb* infection.

### 2.10. Termination of BCG and M. tb Granulomas for CFUs Assay

Termination of granulomas from healthy subjects and individuals with T2DM was performed by collecting and storing the cell-free supernatants and lysing the granulomas using 250 μL of ice cold, sterile 1 × PBS. Cell lysates collected from the wells were vigorously vortexed and then subjected to freeze/thaw cycles to ensure complete lysis of immune cells. The collected lysates and supernatants were then diluted in sterile 1 × PBS and plated on 7H11 medium enriched with ADC to evaluate the survival of mycobacteria inside the granulomas by counting the bacterial colonies.

### 2.11. Hematoxylin and Eosin Staining

Granulomas on cover glasses terminated at 15 days post-infection were fixed with 4% paraformaldehyde (PFA) at room temperature for an hour. Fixed granulomas were washed once with 1 × PBS and stained with Rapid H&E (Scientific R&D Corp, Bay Shore, NY, USA) for 1 min and excess stain was washed with tap water. The cover glasses were inverted and mounted onto slides with mounting media.

### 2.12. Fluorescent Staining

PBMCs (6 × 10^5^/well) plated on a cover-glass positioned in a 24-well tissue culture plate were infected with *M. tb* expressing GFP at an MOI of 0.1:1. Infected cells were treated with lysotracker red DND99 to label acidified compartments within cells and maintained at 37 °C with 5% CO_2_ for 15 days. Granulomas terminated at 15 days post-infection were fixed with 4% PFA at room temperature for an hour. Fixed granulomas on cover-glasses were washed once with 1 × PBS. The cover glasses were inverted and mounted onto slides with mounting media containing DAPI stain to visualize cell nuclei. These slides were sealed with nail polish and observed under fluorescent microscope to quantify colocalization of GFP-expressing bacteria with lysotracker indicative of phagosome acidification.

### 2.13. Quantifying GSH Levels

Measurement of total and oxidized glutathione was performed using the GSH colorimetric assay kit from Arbor Assays (Ann Arbor, MI, USA). rGSH was calculated by subtracting the GSSG from the total GSH. All measurements were corrected for total protein levels.

### 2.14. Quantifying Levels of Malondialdehyde

The malondialdehyde (MDA) assay was used for measurement of oxidative stress. MDA is a byproduct of lipid peroxidation. Once MDA reacts with thiobarbituric acid at 100 °C, a color change occurs, which can be measured colorimetrically at 535 nm. MDA levels were measured using the protocol from the TBARS assay kit from Cayman Chemical. All measurements were corrected for total protein levels.

### 2.15. Quantifying Total Protein Levels

Total protein was measured with a BCA Protein Assay Kit from Thermo Scientific (Rockford, IL, USA).

### 2.16. Assay of Cytokines

Levels of extracellular cytokines such as IL-6, IL-10, IL-12, IFN-γ, and TNF-α, in the granuloma supernatants from healthy subjects and individuals with T2DM were measured by sandwich ELISA. The assay kits were purchased from eBioscience.

### 2.17. Statistical Analysis

Statistical data analyses were performed using GraphPad Prism version 7. Baseline levels of GSH, rGSH, MDA, IL-6, IL-10, IL-12, IFN-γ, and TNF-α were compared between healthy individuals and T2DM group.

## 3. Results

### 3.1. Survival of BCG in 7H9 Media

**Figure 1:**
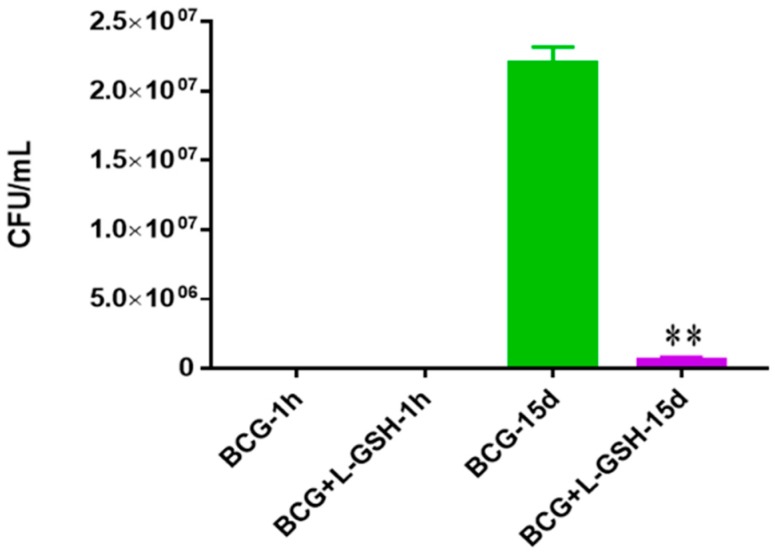
Survival of BCG in 7H9. This experiment was done in order to determine direct killing effects of L-GSH on BCG without the help of immune cells present. M. *bovis* BCG was grown in 7H9 in the presence and absence of L-GSH (40 μM), the same concentration of L-GSH used in treatment of granulomas from healthy and T2DM individuals. There was a significant reduction of bacterial numbers when treated with L-GSH at 15 days. Data represent means ± SE from 2 trials and plating each multiple times. ** *p* < 0.005 when comparing L-GSH treated samples to untreated samples at 15 days.

To determine the survival of the BCG, bacteria were inoculated in 7H9 media over 15 days in the absence of immune cells to test the direct bactericidal effects of the ReadiSorb L-GSH. When treated with L-GSH, the bacterial survival of BCG decreased significantly after 15 days compared to the untreated group (Figure 1).

### 3.2. Hematoxylin and Eosin Staining of Granulomas from Healthy Individuals Infected with BCG

**Figure 2:**
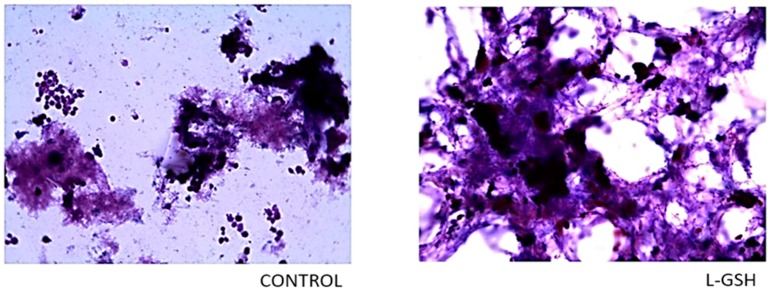
Hematoxylin and eosin staining of granulomas from healthy individuals infected with BCG. Histological staining of granulomas from healthy individuals’ PBMCs infected with BCG showed a more organized structure when treated with L-GSH. Microscopy work was done with a light microscope at 1000 × magnification under oil immersion.

Compared to the slides with no treatment (Figure 2), a noticeable increase in the aggregation of cells treated with L-GSH was observed. An escalation in cell size was also detected, which can be deduced as the increased differentiation of monocytes to macrophages upon direct interaction with the bacteria and lymphocytes and early granuloma formation. These observations indicate that restoring redox homeostasis result in formation of solid and stable granulomas. The monocytes in the control group remained consistent in size and did not form robust aggregations.

### 3.3. Survival of BCG in Granulomas of Healthy Individuals

**Figure 3:**
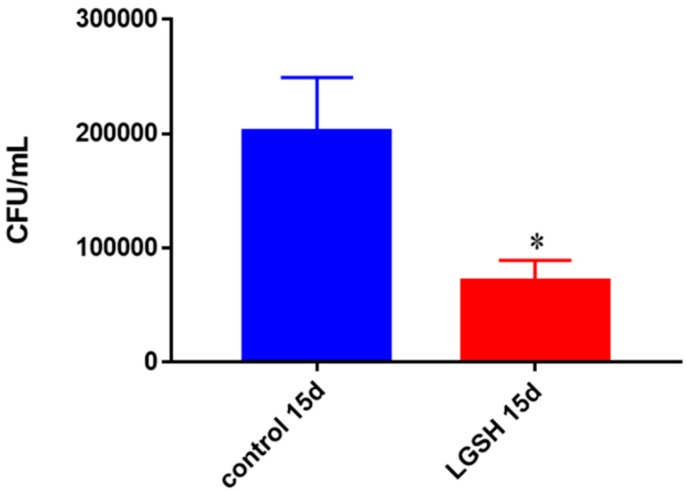
Survival of BCG in in vitro granulomas of healthy individuals. All samples were separated through density dependent centrifugation from peripheral blood of volunteers and PBMCs were isolated after washes with PBS. In each category 6 × 10^4^ bacteria and 6 × 10^5^ immune cells were used for an MOI of 0.1:1. CFU counts of granulomas formed from healthy individuals’ PBMCs showed a decrease at 15 days when treated with L-GSH. Data represent means ± SE from eight healthy individuals. * *p* < 0.05 when comparing L-GSH treated samples to untreated samples at 15 days.

BCG growth was significantly encumbered when treated with L-GSH from healthy immune cells (PBMCs) (Figure 3). There was more than 50% reduction in the survival of BCG in L-GSH-treated granulomas.

### 3.4. IL-6 Levels in Granulomas from Healthy Individuals Infected with BCG

**Figure 4:**
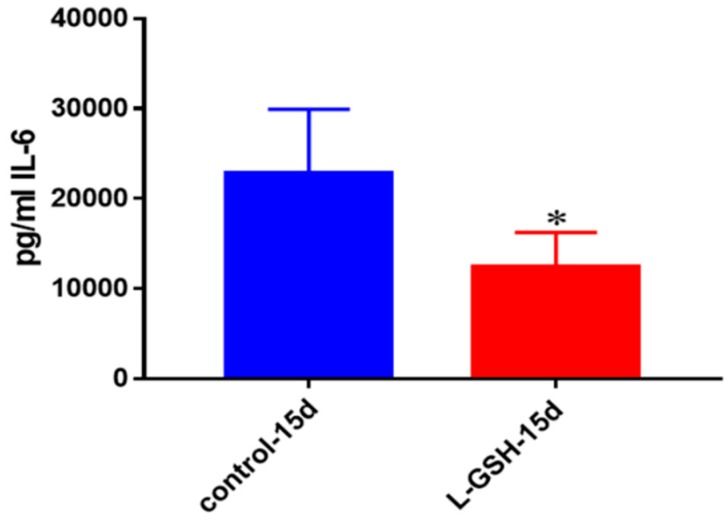
IL-6 levels in granulomas from healthy individuals infected with BCG. Assay of IL-6 was performed using an ELISA Ready-Set-Go kit from eBioscience. There was a two-fold reduction in levels of IL-6 when healthy individuals’ PBMCs were treated with LGSH after infection with BCG. Data represent means ± SE from eight healthy individuals. * *p* < 0.05 when comparing L-GSH treated samples to untreated samples at 15 days.

The production of IL-6, a proinflammatory cytokine released by macrophages and T cell in order to trigger inflammation, was reduced when the granulomas were treated with L-GSH among the healthy individuals (Figure 4). These results indicate that, 15 days post-infection, healthy individuals were able to confine the bacteria within the granulomas and subsequently stimulation of this cytokine declined.

### 3.5. TNF-α Levels in Granulomas from Healthy Individuals Infected with BCG

**Figure 5:**
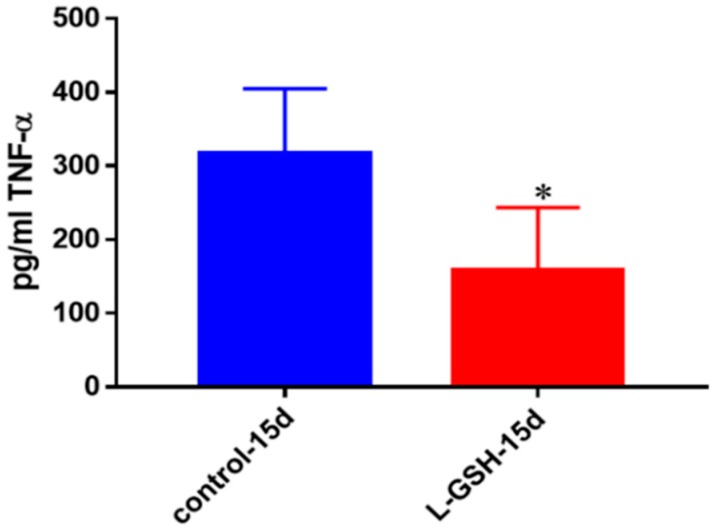
TNF-α levels in granulomas from healthy individuals infected with BCG. Assay of TNF-α was performed using an ELISA Ready-Set-Go kit from eBioscience. There was a significant reduction in levels of TNF-α when healthy individuals’ PBMCs were treated with LGSH after infection with BCG. Data represent means ± SE from eight healthy individuals. * *p* < 0.05 when comparing L-GSH treated samples to untreated samples at 15 days.

L-GSH treatment resulted in decreased levels of TNF-α, an inflammatory cytokine at 15 days post-infection (Figure 5). Although this cytokine is predominantly produced by macrophages, its production has been identified from many other immune cells as well. TNF-α is an important regulator of many types of immune cells, causing cell death or cachexia, a common condition suffered by TB patients that includes weight loss, fatigue, or general weakness [21]. The decreased levels of this cytokine in L-GSH treated granulomas indicate that granuloma formation and *M. tb* reduction play an important function in reducing acute phase reactions, as well as overproduction of proinflammatory cytokines.

### 3.6. IL-10 Levels in Granulomas from Healthy Individuals Infected with BCG

**Figure 6:**
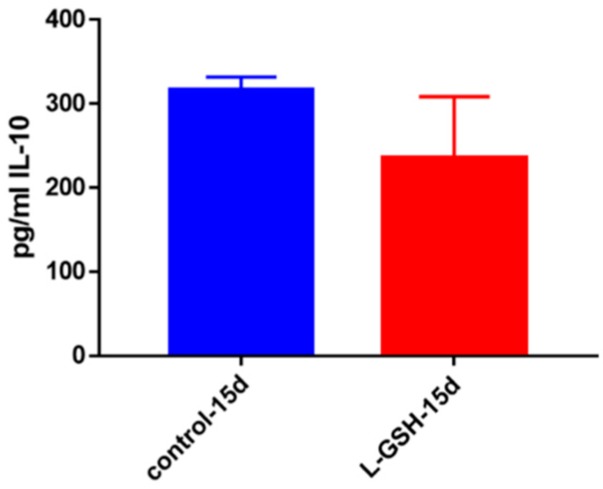
IL-10 levels in granulomas from healthy individuals infected with BCG. Assay of IL-10 was performed using an ELISA Ready-Set-Go kit from eBioscience. Although not significant, there was a reduction in levels of IL-10 when healthy individuals’ PBMCs were treated with L-GSH after infection with BCG. Data represent means ± SE from eight healthy individuals.

IL-10, an anti-inflammatory cytokine, is responsible for the regulation of many proinflammatory cytokines, which are produced in response to bacterial substances like lipopolysaccharides (LPS) [22,23,24,25]. Although not statistically significant, the decreased levels of this cytokine may correlate with downregulation in the inflammatory responses inside the granulomas due to reduced bacterial burden (Figure 6).

### 3.7. MDA Levels in Granulomas from Healthy Individuals Infected with BCG

**Figure 7:**
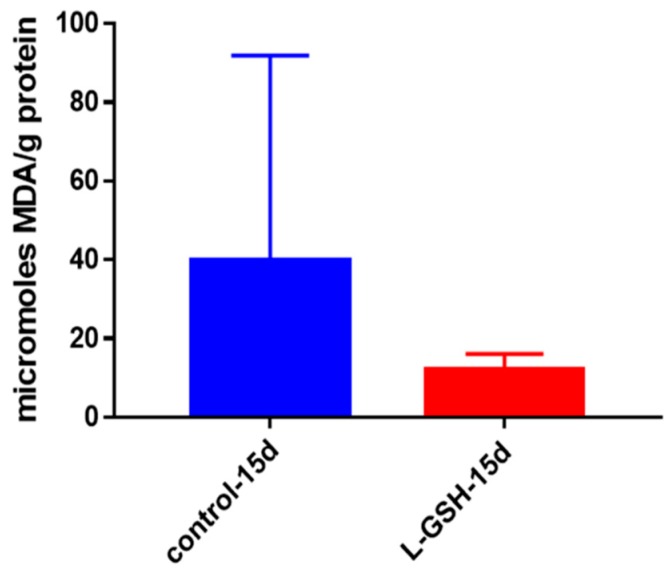
MDA levels in granulomas from healthy individuals infected with BCG. Assay of MDA was performed using a TBARS kit from Cayman Chemical. Although not significant, there was a notable reduction in the levels of MDA or oxidative stress when healthy individuals’ PBMCs were treated with L-GSH after infection with BCG. Data represent means ± SE from eight healthy individuals.

Malondialdehyde (MDA) is a substance measured indicative of lipid peroxidation. Lipid peroxidation is the oxidative degradation of lipids, a process in which free radicals sequester electrons from lipids bound in cell membranes, leading to cellular membrane damage [26]. Although not statistically significant, there was a noticeable decrease in levels of MDA from the granulomas treated with L-GSH (Figure 7). In conjunction with our other findings, the decreased levels of MDA indicate that control over oxidative stress is enhanced after treatment with L-GSH.

### 3.8. GSH Levels in Granulomas from Healthy Individuals Infected with BCG

**Figure 8:**
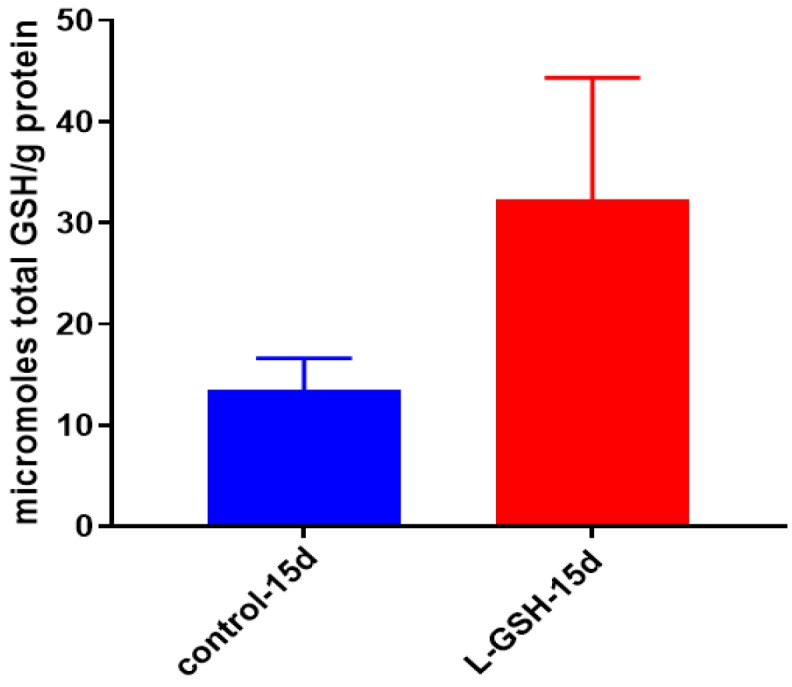
Total GSH levels in granulomas from healthy individuals infected with BCG. GSH assay was performed using a colorimetric assay kit from Arbor Assays. Corrections were made to total protein measured by BCA Protein Assay Kit from Thermo Scientific. Both the GSH and total protein levels were measured in lysates excluding any supernatants from samples. There was a notable increase in the levels of GSH in L-GSH-treated granulomas. Data represent means ± SE from eight healthy individuals.

When the granuloma lysates were measured, the levels of GSH displayed a marked increase when post treated with L-GSH (Figure 8). Since the GSH used to treat the samples are encapsulated within liposomes, and the measurement is taken only from the sample lysates, the measured results are indicative of only the GSH that has been taken up by the cells. GSH is used in the neutralization of free radicals that cause oxidative stress; the increase of GSH (Figure 8) is in parity with the aforementioned lowered oxidative stress observed (Figure 7).

### 3.9. Survival of M. tb in 7H9 Media

**Figure 9:**
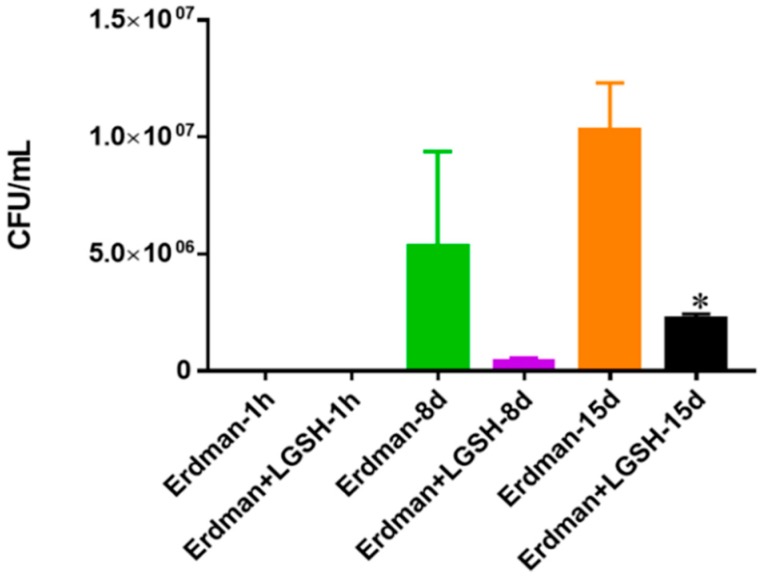
Survival of Erdman strain of *M. tb* in 7H9 media. This experiment was done in order to determine direct killing effects of L-GSH on Erdman strain of *M. tb* without the help of immune cells present. *M. tb* was grown in 7H9 in the presence and absence of L-GSH (40 μM), the same concentration of L-GSH used in treatment of granulomas from healthy and T2DM individuals. There was a significant reduction in the *M. tb* numbers when treated with L-GSH at 15 days and a notable decrease at eight days. Data represent means ± SE from two trials and plating each multiple -times. * *p* < 0.05 when comparing L-GSH treated samples to untreated samples at 15 days.

The growth of the Erdmann strain of *M. tb* was achieved in 7H9 media. Within eight days there was an observable decrease in the survival of the bacteria after treatment with L-GSH. After 15 days in media the results presented that the number of bacteria had significantly decreased after treatment with L-GSH compared to the control with no additive supplementation (Figure 9). These results are comparable to the bacterial conditions seen from the BCG experiment (Figure 1) after treatment with L-GSH in the absence of immune cells. While the growth of BCG after 15 days was twice as much as the virulent Erdman strain, this may be due to the slower growth tendency of the *M. tb* strain, as was also observed when anticipating countable colonies to appear.

### 3.10. Hematoxylin and Eosin Staining of THP-1 Cells Infected with M. tb

**Figure 10:**
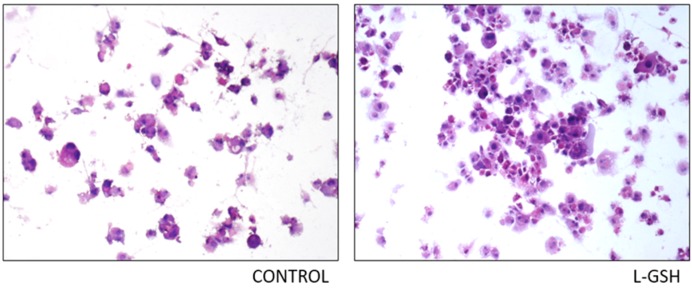
Hematoxylin and eosin staining of THP-1 cells infected with Erdman strain of *M. tb*: Histological staining of granulomas from THP-1 cells infected with *M. tb* showed a more organized structure when treated with LGSH. Microscopy work was done with a light microscope at 100× magnification.

The histological staining of THP-1 cells with and without supplementation of L-GSH displayed greater cellular aggregation after L-GSH treatment (Figure 10). The observed untreated cells have the tendency to be more scattered, with little to no solid granuloma formation.

### 3.11. Survival of Erdman in THP-1 Cells

**Figure 11:**
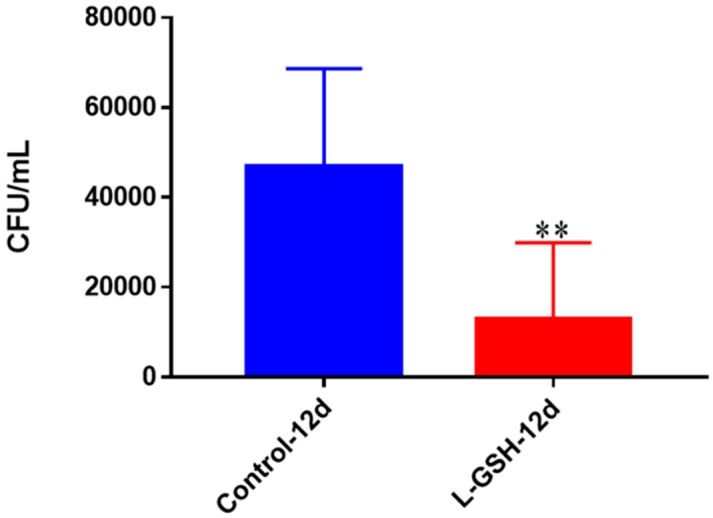
Survival of *M. tb* in THP-1 cells. THP-1 cells were cultured in a medium of RPMI and 10% FBS, and allowed to differentiate into macrophages by addition of PMA at a concentration of 10 ng/mL. *M. tb*-infected macrophages (2 × 10^5^/well) were either untreated or treated with L-GSH (40 μM). There was a significant reduction in *M. tb* numbers when THP-1 cells were treated with L-GSH. Data represent means ± SE from six trials, plating each multiple times. ** *p* < 0.005 when comparing L-GSH treated samples to untreated samples at 12 days.

Although the human granulomas were allowed to remain viable for 15 days, the THP-1 cells appeared to deteriorate when left that long. For this reason, we decided to terminate the THP-1 cells after 12 days, which appeared to be the zenith of their viability. Twelve days post-infection, the THP-1 macrophages treated with L-GSH exhibited a significant reduction in the quantity of persisting bacteria (Figure 11).

### 3.12. TNF-α Levels in THP-1 Cells Infected with M. tb

**Figure 12:**
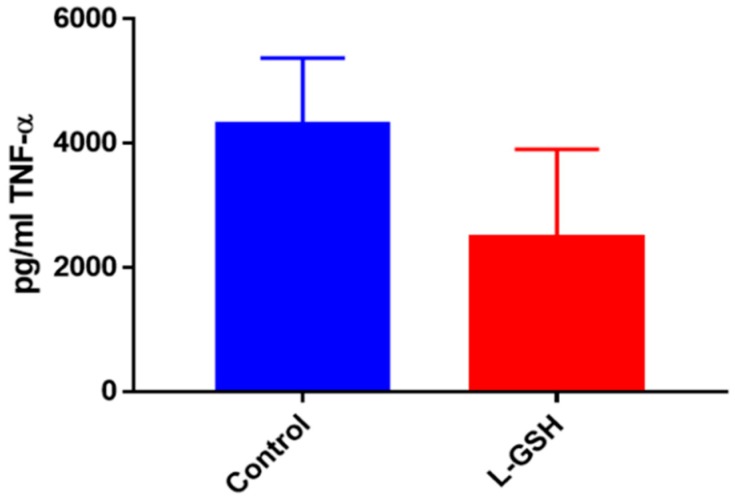
TNF-α levels in THP-1 cells infected with *M. tb*. Assay of TNF-α was performed using an ELISA Ready-Set-Go kit from eBioscience. There was an observable reduction in levels of TNF-α when THP cells were treated with LGSH after infection with *M. tb* after 12 days. Data represent means ± SE from six trials plating each multiple -times.

TNF-α levels revealed a decrease among the THP-1 cells treated with L-GSH (Figure 12). Although the levels of this proinflammatory cytokine were 10-fold higher in the THP-1 samples, this trend is comparable to granulomas formed from PBMCs from healthy human individuals (Figure 5).

### 3.13. IL-10 Levels in THP-1 Cells Infected with M. tb

**Figure 13:**
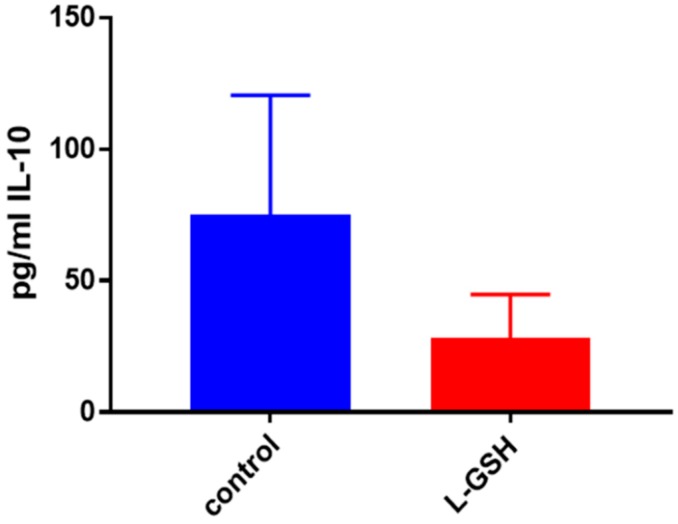
IL-10 levels in THP -1 cells infected with *M. tb*. Assay of IL-10 was performed using an ELISA Ready-Set-Go kit from eBioscience. There was a drastic reduction in levels of IL-10 when THP-1 cells were treated with LGSH after infection with Erdman at 12 days. Data represent means ± SE from six trials, plating each multiple- times.

L-GSH treatment resulted in downregulation in the levels of IL-10, and the trend was similar to that of the granulomas from healthy subjects (Figure 6 and Figure 13, respectively). However, there was a three-fold increase in the levels of this anti-inflammatory cytokine in untreated subjects. BCG granulomas from the healthy subjects were compared to the untreated THP-1 cells.

### 3.14. IL-6 Levels in THP-1 Cells Infected with M. tb

**Figure 14:**
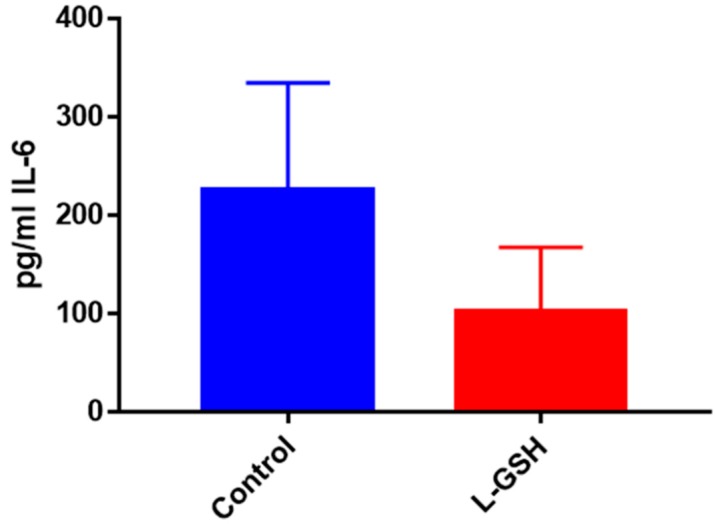
IL-6 levels in THP-1 cells infected with Erdman Assay of IL-6 was performed using an ELISA Ready-Set-Go kit from eBioscience. There was a notable reduction in the levels of IL-6 when THP cells were treated with LGSH after infection with Erdman at 12 days. Data represent means ± SE from six trials, plating each multiple- times.

The levels of IL-6, a proinflammatory cytokine, presented a marked decrease when the THP-1 cells were treated with L-GSH (Figure 14). These results indicate L-GSH’s ability to provide additional support to the innate immune system by maintaining homeostasis and a functional environment for host cells to keep bacterial growth under control.

### 3.15. Hematoxylin and Eosin Staining of Granulomas from Healthy Individuals Infected with M. tb Compared to Granulomas from T2DM Individuals

**Figure 15:**
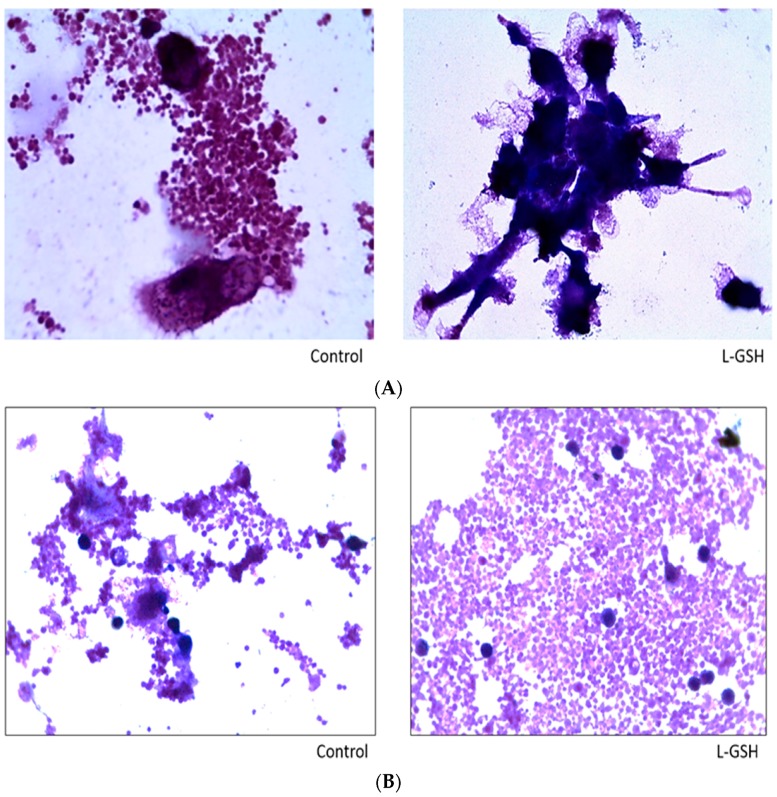
(**A**) Hematoxylin and Eosin staining of granulomas from healthy individuals infected with *M. tb*. There was an aggregation of cells in both treated and untreated groups of granulomas formed by PBMCs of healthy individuals infected with Erdman strain of *M. tb*. But there was an apparent increase in the size of granulomas when treated with L-GSH; (**B**) Hematoxylin and Eosin staining of granulomas from T2DM individuals infected with *M. tb*. Granulomas were formed from PBMCs from T2DM patients. There was a great extent of cell aggregation when treated with L-GSH versus untreated. Microscopy work was done with a light microscope at 1000× magnification under oil immersion.

Both L-GSH treated and untreated groups formed aggregations of cellular granulomas from the PBMCs of healthy individuals infected with the Erdman strain of *M. tb* (Figure 15A). However, there was a discernible increase in cell size after treatment with L-GSH. The observed increase in cellular size and membrane projections are on par with the cells’ differentiation to macrophages when activated by an external stimulus such as *M. tb*. Histological staining of granulomas formed by PBMCs from T2DM individuals displayed an interesting trend (Figure 15B). The granulomas formed by the PBMCs from healthy individuals (Figure 15A) exhibited larger cell aggregates when treated with L-GSH. We identified this change as the result of more activated macrophages and other immune cells within a granuloma; however, the T2DM patients did not display these larger cell clusters. Additionally, we observed much more aggregation of these smaller cells when treated with L-GSH.

### 3.16. Survival of M. tb in Untreated Granulomas of Healthy Individuals

**Figure 16:**
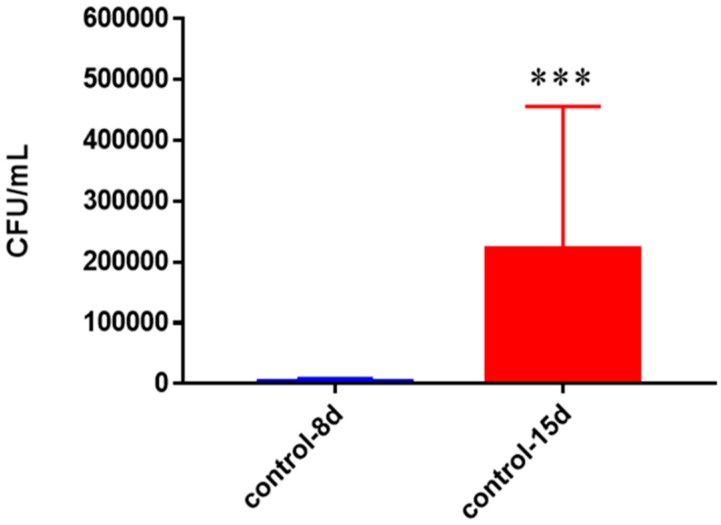
Survival of *M. tb* in untreated granulomas of healthy individuals. All samples were separated through density dependent centrifugation from peripheral blood of volunteers and PBMCs were isolated after washes with PBS. In each category 6 × 10^4^ bacteria and 6 × 10^5^ immune cells were used for an MOI of 0.1:1. CFU counts of granulomas formed from healthy individuals’ PBMCs showed a statistically significant increase at 15 days when left untreated. Data represent means ± SE from eight healthy individuals. *** *p* < 0.0005 when comparing samples at eight days and 15 days.

In healthy subjects, a substantial growth of *M. tb* was observed with a statistically significant increase in the bacterial numbers between eight days and 15 days when left untreated. By day 15 the *M. tb* numbers had increased more than 7-fold (Figure 16).

### 3.17. Quantification of M. tb within Acidified Compartments in Untreated Granulomas from Healthy Individuals

**Figure 17:**
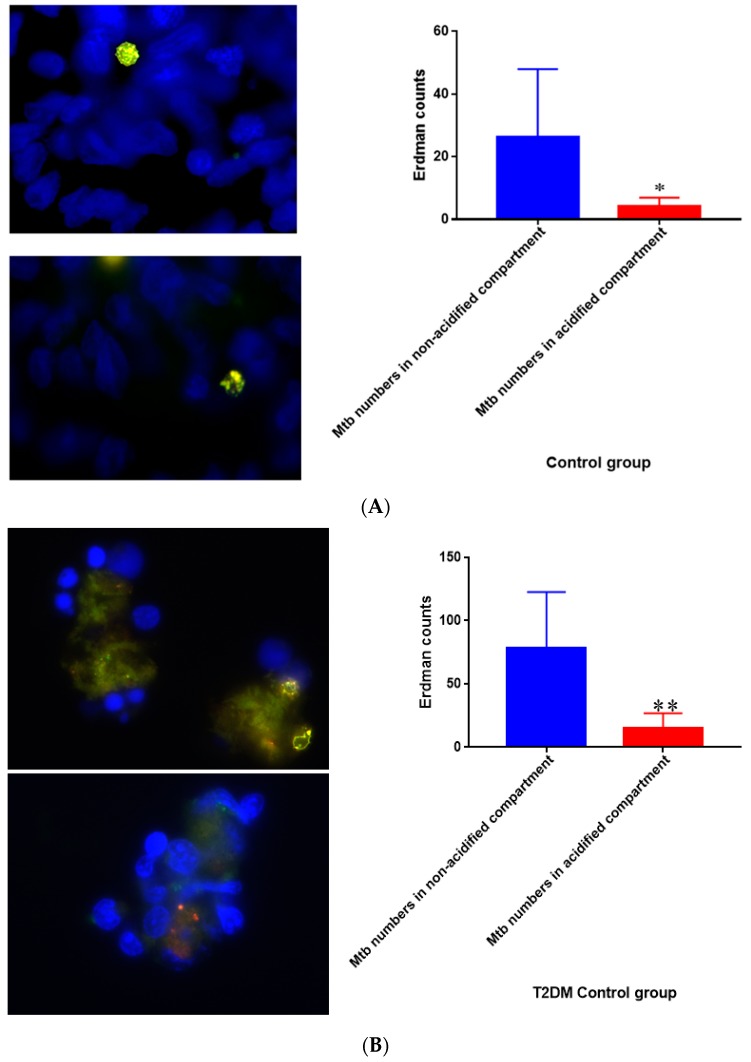
(**A**) Quantification of *M. tb* within acidified compartments in untreated granulomas of healthy individuals. GFP-labeled *M. tb* was used for infection. Granulomas were labeled with lysotracker red DND99, which gets trapped within the acidified compartments, as well as DAPI for nucleus staining. When untreated, at 15 days granulomas showed significantly less *M. tb* in acidified compartments compared to the bacteria present in non-acidified compartments; (**B**) Untreated granulomas of T2DM individuals. Yellow areas correspond to acidified compartments with GFP-labeled bacteria present. Data represent means ± SE from eight healthy individuals. * *p* < 0.05 when comparing bacterial numbers in acidified versus non-acidified compartments. ** *p* < 0.005 when comparing bacterial numbers in acidified versus non-acidified compartments.

Quantification of *M. tb* within acidified compartments of macrophages was accomplished with the use of DAPI and lysotracker labeling of acidified compartment in cells within granulomas. The Erdman strain of *M. tb* used in our experiments is conjugated with GFP, which allows the bacteria to be labeled. When the bacteria are used in conjunction with a lysotracker that labels acidified compartments, an overlap can be observed in yellow. When left untreated, the 15-day granulomas displayed a decrease in the amount of *M. tb* within acidified compartments compared to the bacteria present in non-acidified compartments (Figure 17A). This measurement was taken by counting yellow (or red) fluorescence indicative of the bacteria present in acidified compartments and comparing them to green areas, revealing *M. tb* existing in non-acidified compartments. Similar trends were observed in T2DM granulomas, but the bacterial numbers were a lot higher in comparison (Figure 17B). Acidified compartments refer to phagosomes that have fused with an acidic lysosome to cause the breakdown of the contents inside the compartment. Non-acidified compartments refer to phagosomes that contain bacteria but have not fused with a lysosome.

### 3.18. Survival of M. tb in Untreated and L-GSH-Treated Granulomas from Healthy Individuals and Granulomas of T2DM Individuals

**Figure 18:**
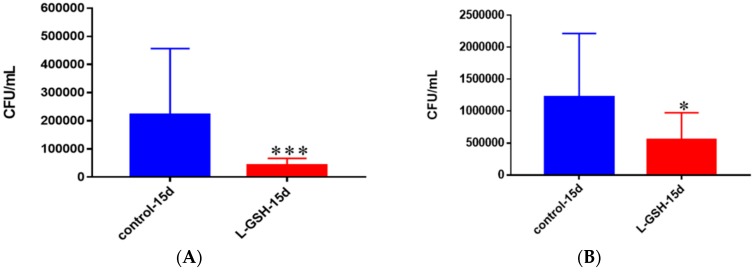
(**A**) Survival of *M. tb* in untreated and L-GSH granulomas of healthy individuals. All samples were separated through density dependent centrifugation from peripheral blood of volunteers and PBMCs were isolated after washes with PBS. In each category 6 × 10^4^ bacteria and 6 × 10^5^ immune cells were used for an MOI of 0.1:1. CFU counts of granulomas formed from healthy individuals showed a statistically significant decrease at 15 days when treated with L-GSH; (**B**) Survival of *M. tb* in granulomas of T2DM individuals. All samples were separated through density-dependent centrifugation from peripheral blood of volunteers and PBMCs were isolated after washes with PBS. In each category 6 × 10^4^ bacteria and 6 × 10^5^ immune cells were used for an MOI of 0.1:1. CFU counts of granulomas formed from individuals with T2DM showed a statistically significant decrease at 15 days when treated with L-GSH. Data represent means ± SE from eight healthy individuals and six T2DM individuals. *** *p* < 0.0005 when comparing samples left untreated and treated with L-GSH for 15 days. * *p* < 0.05 when comparing untreated samples with samples treated with L-GSH at 15 days.

Granulomas from healthy individuals were significantly more successful in controlling *M. tb* infection after supplementation with L-GSH than without the treatment (Figure 18A). This is represented by the 5-fold lower bacterial detection observed in the granulomas that were treated with L-GSH. With L-GSH treatment the granulomas formed from T2DM PBMCs, displayed a 50% reduction in *M. tb* compared to the untreated group (Figure 18B). A comparison between bacterial survival in granulomas of T2DM individuals and healthy individuals (Figure 17A,B) shows that, although there was a similar trend of bacterial reduction when treated with L-GSH, the magnitude of *M. tb* growth was much greater in T2DM individuals than it was in healthy individuals. In fact, when left untreated the T2DM samples had *M. tb* numbers that were elevated 5-fold, and roughly 10-fold greater for the samples treated with L-GSH.

### 3.19. Quantification of M. tb within Acidified Compartments in Granulomas of Healthy Individuals Treated with L-GSH

**Figure 19:**
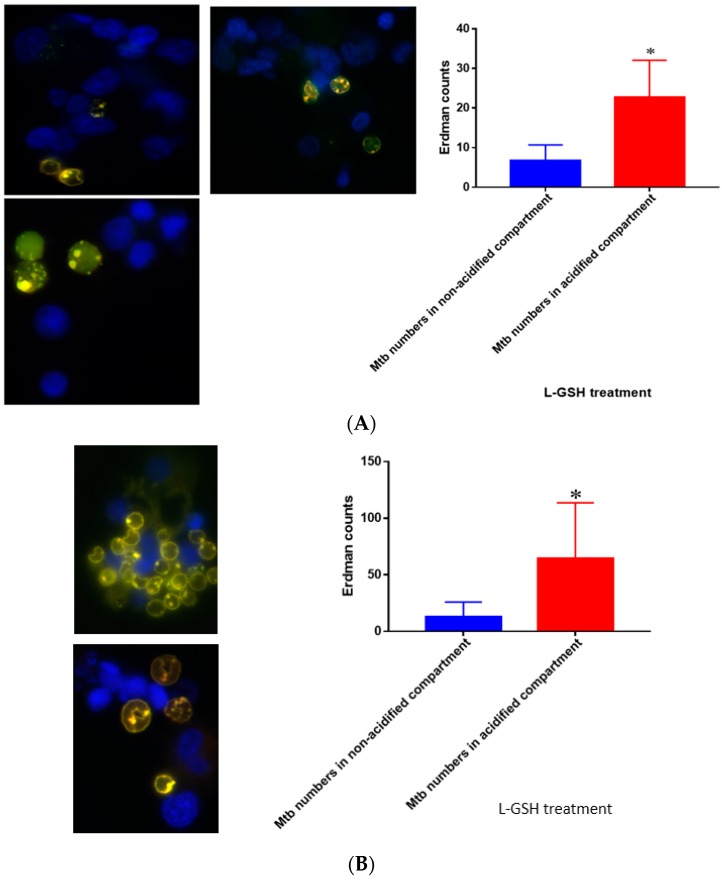
(**A**) Quantification of Erdman within acidified compartments in PBMCs of healthy individuals treated with L-GSH. Green fluorescent protein (GFP)-labeled *M. tb* was used with lysotracker red DND99, which labeled acidified compartments, as well as DAPI for nucleus staining. When treated with L-GSH, granulomas showed an increase in bacterial number in acidified compartments compared to the bacteria present in non-acidified compartments. Yellow areas correspond to acidified compartments with GFP-labeled bacteria; (**B**) fluorescent staining of granulomas from T2DM individuals infected with *M. tb*. GFP-labeled *M. tb* was used with lysotracker red DND99, which labeled acidified compartments, as well as DAPI for nucleus staining. When treated with L-GSH, granulomas showed increased numbers of *M. tb* in acidified compartments compared to the bacteria present in non-acidified compartments. Yellow areas correspond to acidified compartments with GFP labeled bacteria present. Data represent means ± SE from five T2DM individuals and eight healthy individuals. * *p* < 0.05 when comparing bacterial numbers in acidified versus non-acidified compartments.

L-GSH treatment of granulomas from healthy individuals resulted in an increase in the number of *M. tb* within acidified compartments compared to the control (Figure 19A). These results are consistent with our previous findings, which suggest that L-GSH plays an integral role in successful phagosome–lysosome fusion within macrophages, an essential component of *M. tb* control. When treated with L-GSH, there was a significant increase in *M. tb* within the acidified compartments of the granulomas compared to the bacteria present in non-acidified compartments for individuals with T2DM (Figure 19B). Indicative are the yellow areas, which correspond to acidified compartments with GFP-labeled bacteria present.

### 3.20. Total GSH Levels in M. tb-Infected Granulomas from Healthy Individuals

**Figure 20:**
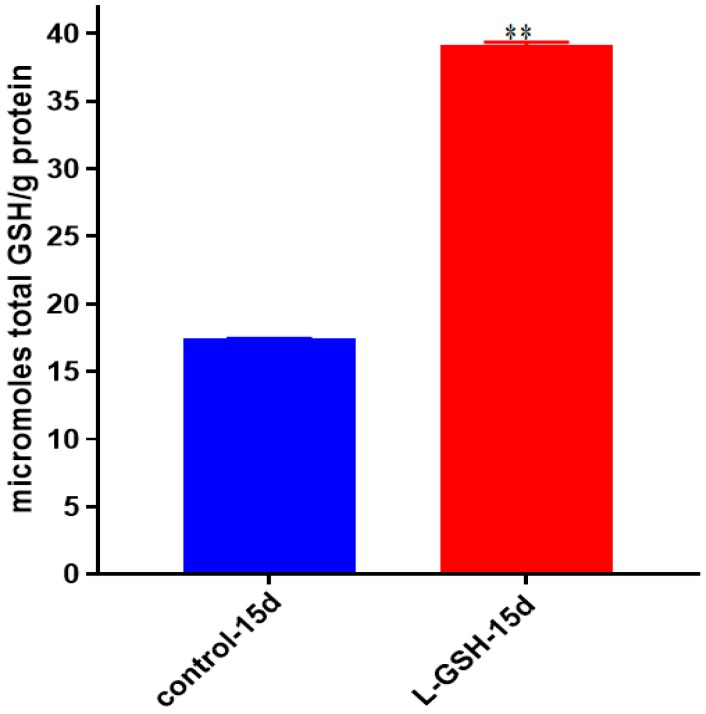
Total GSH levels in granulomas from healthy individuals infected with *M. tb.* GSH assay was performed using a colorimetric assay kit from Arbor Assays. Corrections were made to total protein measured by BCA Protein Assay Kit from Thermo Scientific. Both the GSH and total protein levels were measured in lysates excluding any supernatants from samples. Once treated with L-GSH, granulomas from healthy individuals had a statistically significant increase in cellular levels of GSH. Data represent means ± SE from eight healthy individuals. ** *p* < 0.005 when comparing GSH levels in control and L-GSH treated groups.

GSH levels were measured in the granuloma lysates from healthy individuals at 15 days post-*M. tb* infection with and without treatment of L-GSH. Similar to previous trends, higher levels of GSH were detected after L-GSH treatment (Figure 20). The concentration of GSH present in untreated granulomas infected with *M. tb* was comparable to untreated granulomas infected with BCG (Figure 8).

### 3.21. IFN- γ Levels in M. tb Granulomas from Healthy Individuals

**Figure 21:**
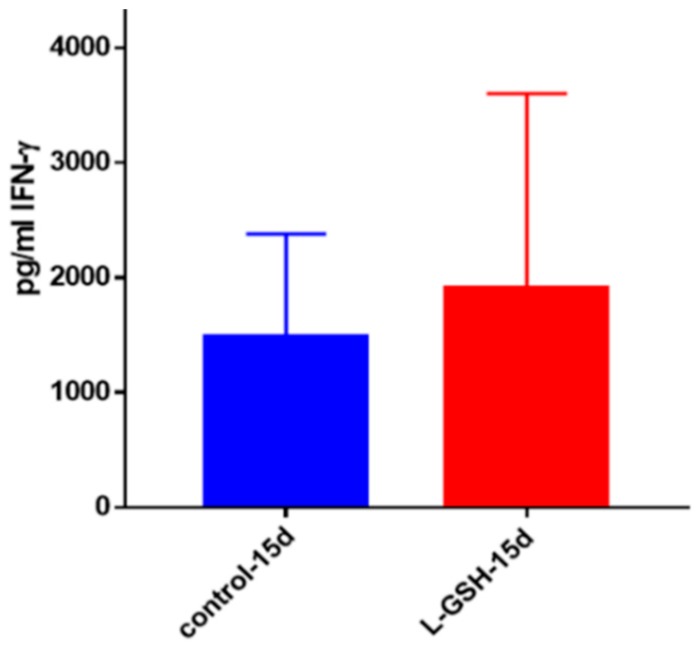
IFN-γ levels in *M. tb*-infected granulomas from healthy individuals. Assay of IFN-γ was performed using an ELISA Ready-Set-Go kit from eBioscience. There was a modest increase in levels of IFN-γ measured in supernatants of granulomas from healthy subjects. Data represent means ± SE from eight healthy individuals.

IFN-γ is a proinflammatory cytokine pivotal for the activation of macrophages. IFN-γ possesses immunostimulatory effects that can induce effector mechanisms in macrophages leading to the control of *M. tb* infection. The levels of IFN-γ measured in the supernatants of the L-GSH-treated granulomas from healthy subjects showed a marked increase compared to the untreated controls (Figure 21).

### 3.22. TNF- α Levels in M. tb Granulomas from Healthy Individuals and T2DM Individuals at 15 Days

**Figure 22:**
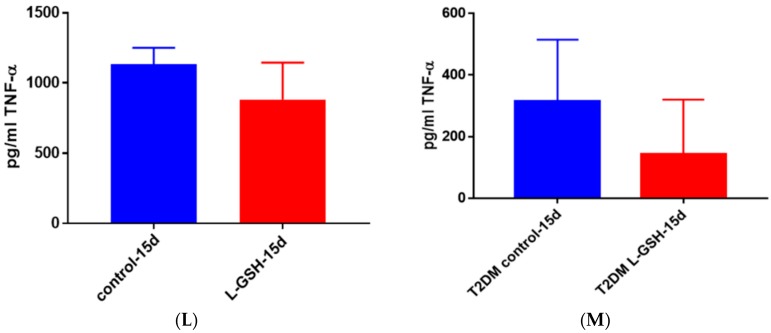
(**A**) TNF-α levels in *M. tb*-infected granulomas from healthy individuals at 15 days. There was a slight reduction in levels of TNF-α when healthy individuals. Granulomas were treated with LGSH after infection with Erdman after 15 days; (**B**) TNF-α levels in *M. tb*-infected granulomas from individuals with T2DM individuals. Assay of TNF-α was performed using an ELISA Ready-Set-Go kit from eBioscience. There was a reduction in levels of TNF-α when T2DM individuals. Granulomas were treated with LGSH after infection with Erdman. Data represent means ± SE from six T2DM individuals and eight healthy individuals.

At 15 days, there was a modest decrease in the levels of TNF-α in *M. tb* granulomas from healthy individuals (Figure 22A). TNF-α, being an immunomodulatory cytokine, appears to retain stable levels once a granuloma has been established amongst healthy individuals. The TNF-α levels in THP-1 cells infected with *M. tb* (Figure 12), as well as in T2DM granulomas (Figure 22B) exhibited a decrease by 15 days. This led us to believe that, by 15 days, once a stable granuloma has been formed among healthy individuals, there is no longer a need for the acute phase reaction that is generated by TNF-α and subsequently the cytokine production returns to normal levels. There was a noticeable decrease in the levels of TNF-α in granulomas of T2DM individuals treated with L-GSH (Figure 22B). This trend is comparable to the TNF-α levels detected from the granulomas from healthy individuals at eight-day time points. This trend further suggests that, although constant levels of TNF-α are required for early granuloma formation, the levels of TNF-α seemed to decrease after successful control of *M. tb* within granulomas has been accomplished, and subsequently this cytokine is no longer needed in great quantity for the maintenance of the granulomas.

### 3.23. TNF-α Levels in Healthy Individuals Infected with Erdman at Eight Days

**Figure 23:**
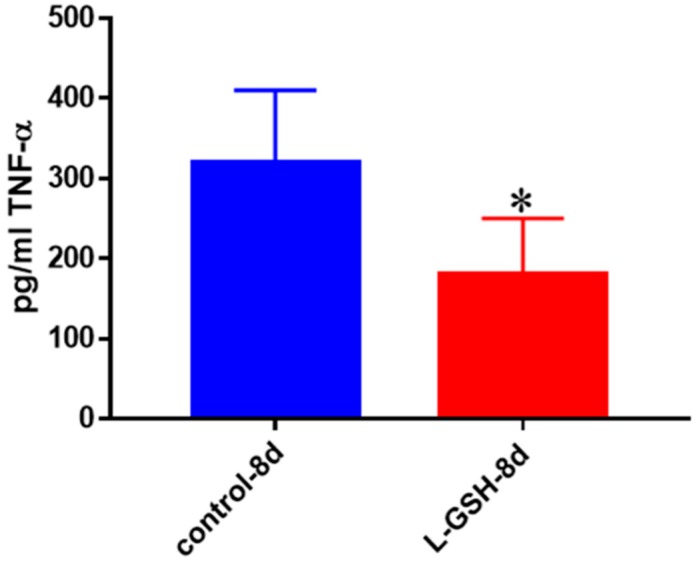
TNF-α levels in *M. tb*-infected granulomas from healthy individuals at eight days. Assay of TNF-α was performed using an ELISA Ready-Set-Go kit from eBioscience. There was a statistically significant reduction in levels of TNF-α when healthy individuals’ granulomas were treated with LGSH after infection with Erdman after eight days. Data represent means ± SE from eight healthy individuals. * *p* < 0.05 when comparing TNF-α levels in control and L-GSH-treated groups.

L-GSH treatment caused a significant decrease in the levels of TNF-α levels in *M. tb*-infected granulomas from healthy subjects at eight days post-infection (Figure 23). These results, when to compared to the findings at 15-day time-point (Figure 22A) indicate that the production of TNF-α at eight-days post-infection is three times lower than at the 15-day time point. Furthermore, L-GSH has significant downregulating effects on the production of TNF-α at day 8 compared to 15 days post-infection.

### 3.24. IL-6 Levels in M. tb-Infected Granulomas from Healthy Individuals

**Figure 24:**
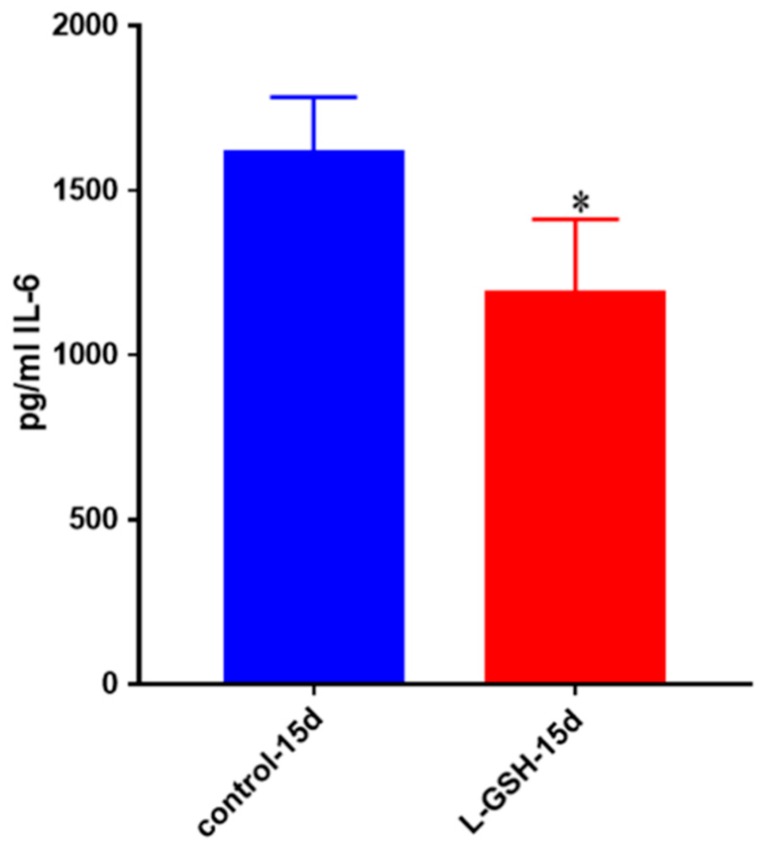
IL-6 levels in *M. tb*-infected granulomas from healthy individuals. Assay of IL-6 was performed using an ELISA Ready-Set-Go kit from eBioscience. There was a significant reduction in levels of IL-6 when healthy individuals’ granulomas were treated with LGSH after infection with Erdman. Data represent means ± SE from eight healthy individuals. * *p* < 0.05 when comparing L-GSH treated samples to untreated samples at 15 days.

IL-6, another pro-inflammatory cytokine, was also shown to be significantly decreased after treatment with L-GSH among the healthy individuals (Figure 24). This trend is comparable to the levels of IL-6 seen in the BCG infected healthy granulomas (Figure 4) as well as the Erdman-infected THP cells (Figure 14). However, the levels of IL-6 from THP cells exhibited a 100-fold decrease when compared to the levels from the BCG-granulomas from healthy individuals, and a 10-fold decrease when compared to *M. tb* granulomas from healthy individuals.

### 3.25. IL-10 Levels in M. tb-Infected Granulomas from Healthy Individuals

**Figure 25:**
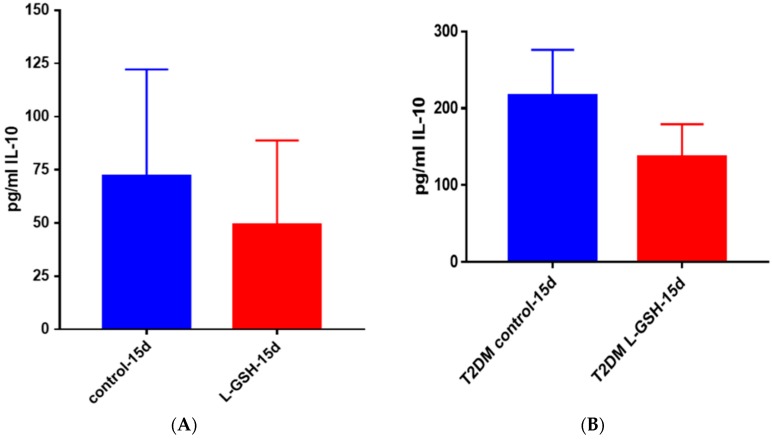
(**A**) IL-10 levels in *M. tb*-infected granulomas from healthy individuals. There was a reduction in levels of IL-10 when healthy individuals; (**B**) IL-10 levels in *M. tb*-infected granulomas from individuals with T2DM. Assay of IL-10 was performed using an ELISA Ready-Set-Go kit from eBioscience. Although not significant, there was a reduction in levels of IL-10 when T2DM individuals. Granulomas were treated with LGSH after infection with Erdman. Data represent means ± SE from six T2DM individuals and eight healthy individuals.

There was reduction in the levels of IL-10 in L-GSH-treated granulomas compared to the untreated granulomas (Figure 25A). The IL-10 trend seemed to stay consistent throughout this study. The levels of IL-10 in THP-1 cells (Figure 13) and healthy granulomas (Figure 25A) infected with *M. tb*, were three times lower than the BCG granulomas from healthy subjects (Figure 6) and *M. tb*-infected granulomas from individuals with T2DM (Figure 25B). Although the IL-10 trends were similar in each experiment in this study, a decrease was observed after supplementation with L-GSH (Figure 25B), the levels of this cytokine were also comparable in magnitude to BCG granulomas from healthy individuals (Figure 6).

## 4. Discussion

*M. tb* infection is acquired through inhalation of bacteria via aerosolized droplets. Upon inoculation of the lungs, bacteria have one of three fates: they are destroyed by host defense mechanisms, leading to no disease; the host immune responses leads to LTBI; or the pathogen can subvert the host defense mechanisms, leading to active TB [27].

In 90% of individuals infected with *M. tb*, the host defense mechanisms will mount an adequate immune response to render the bacteria latent by way of granuloma formation. A granuloma is an accumulation of activated macrophages, epithelioid histiocytes, giant cells, and subsets of T lymphocytes that encapsulate the bacteria and maintain it in a localized region within the lungs, thereby preventing dissemination [28]. Individuals with LTBI are healthy and asymptomatic. Numerous regulatory cytokines and chemokines are involved in the recruitment and formation of the granuloma, necessitating a functioning immune system [29].

In immunocompromised individuals, the LTBI can overcome the host defense mechanisms and disseminate throughout the body, causing active infection. The chronic oxidative stress, redox imbalance, and inflammation that are associated with diseases like HIV and diabetes often lead to inappropriate immune responses, putting the individual at increased risk of *M. tb* infection and reactivation of LTBI [30].

In this study we show T2DM patients’ decreased control over bacterial load. When the PBMCs of an individual with T2DM were introduced to infection (Figure 18B), bacterial numbers were 6-fold higher than when a healthy individual’s PBMCs were introduced to infection (Figure 18A).

GSH, a natural antioxidant present in most of our cells, plays a critical role in maintaining redox homeostasis. Previous studies from our laboratory have demonstrated that levels of intracellular GSH are significantly compromised in HIV-positive subjects and in individuals with T2DM due to compromised levels of enzymes involved in the synthesis of GSH [15,19,20,31,32,33]. Further studies demonstrated that supplementation of HIV-positive subjects with L-GSH for three months restored the levels of GSH, induced cytokine and redox balance, and improved intracellular control of *M. tb* infection [19,32].

This study tested the effects of L-GSH in improving the granulomatous response to BCG (Figure 1) and *M. tb* (Figure 9). We found that L-GSH has direct antimycobacterial effects and was able to significantly reduce the viability of both BCG and Erdman strain of *M. tb*, as quantified by measuring colony-forming units (CFUs). Although both BCG and *M. tb* strain survivability were reduced with L-GSH supplementation, we observed a more significant decrease in BCG survivability compared to the Erdman strain. This is possibly due to the fact that BCG is an attenuated strain of *M. tb*, readily used for vaccination, whereas the Erdman strain is a virulent strain of *M. tb* (more virulent than H37Rv)*.*

L-GSH treatment of BCG granulomas resulted in a significant reduction in the levels of proinflammatory cytokines such as IL-6 and TNF-α (Figure 4 and Figure 5), and a decrease in oxidative stress (Figure 7). L-GSH treatment of BCG granulomas also restored the levels of GSH (Figure 8). These results illustrate the efficacy of L-GSH in modulating the immune responses in the granulomas derived from healthy subjects, leading to improved control of BCG infection.

Studies using in vitro granulomas provided us with important information on the overall collective immune responses (innate and adaptive) against mycobacterial infection. However, to determine the effects of L-GSH in specifically altering the innate immune responses against mycobacterial infection we used THP-1 cells, since macrophages provide first-line defense against *M. tb* infection [34].

In THP-1 cells infected with the Erdman strain of *M. tb*, we observed similar trends of cytokines but IL-6 (Figure 14) was reduced 100-fold compared to healthy individuals’ PBMCs infected with BCG (Figure 4).

In granulomas achieved from healthy individuals’ PBMCs infected with Erdman, we found that more *M. tb* were localized in acidified compartments after treatment with L-GSH. This is a promising find for future studies on the pathway *M. tb* utilizes to survive in macrophages by blocking phagosome–lysosome fusion.

When compared to healthy individuals, T2DM individuals were not as successful at inhibiting the growth and replication of *M. tb*. Observation of healthy (Figure 18A) and T2DM controls (Figure 18B) showed the greater ability of healthy individuals to control bacterial load after infection. After treatment with L-GSH, both treatment groups showed a decrease in CFUs, but healthy individuals showed a stronger immunological response.

L-GSH also facilitated granulomatous control of *M. tb* at a higher magnitude in healthy individuals. *M. tb* numbers were reduced by 75% in healthy individuals, whereas in T2DM they decreased by 60%.

From our results we can conclude that L-GSH helps control *M. tb* numbers and maintain a stable environment for granuloma formation and maintenance by providing help to the immune system by cytokine balance as well as direct mycobacterial killing.
